# Identification of Cognitive Dysfunction in Patients with T2DM Using Whole Brain Functional Connectivity

**DOI:** 10.1016/j.gpb.2019.09.002

**Published:** 2019-11-28

**Authors:** Zhenyu Liu, Jiangang Liu, Huijuan Yuan, Taiyuan Liu, Xingwei Cui, Zhenchao Tang, Yang Du, Meiyun Wang, Yusong Lin, Jie Tian

**Affiliations:** 1 CAS Key Laboratory of Molecular Imaging, Institute of Automation, Chinese Academy of Sciences, Beijing 100190, China; 2 School of Computer and Information Technology, Beijing Jiaotong University, Beijing 100044, China; 3 Department of Endocrinology and Metabolism, Henan Provincial People’s Hospital & the People’s Hospital of Zhengzhou University, Zhengzhou 450003, China; 4 Department of Radiology, Henan Provincial People’s Hospital & the People’s Hospital of Zhengzhou University, Zhengzhou 450003, China; 5 Cooperative Innovation Center for Internet Healthcare & School of Software, Zhengzhou University, Zhengzhou 450003, China; 6 School of Mechanical, Electrical & Information Engineering, Shandong University (Weihai), Weihai 264209, China; 7 University of Chinese Academy of Sciences, Beijing 100080, China; 8 Beijing Advanced Innovation Center for Big Data-Based Precision Medicine, School of Medicine, Beihang University, Beijing 100191, China; 9 Engineering Research Center of Molecular and Neuro Imaging of Ministry of Education, School of Life Science and Technology, Xidian University, Xi’an 710126, China

**Keywords:** Type 2 diabetes mellitus, Resting state functional connectivity, Elastic net, Support vector machines, MoCA

## Abstract

Majority of **type 2 diabetes mellitus** (T2DM) patients are highly susceptible to several forms of cognitive impairments, particularly dementia. However, the underlying neural mechanism of these cognitive impairments remains unclear. We aimed to investigate the correlation between whole brain resting state functional connections (RSFCs) and the cognitive status in 95 patients with T2DM. We constructed an **elastic net** model to estimate the Montreal Cognitive Assessment (**MoCA**) scores, which served as an index of the cognitive status of the patients, and to select the RSFCs for further prediction. Subsequently, we utilized a machine learning technique to evaluate the discriminative ability of the connectivity pattern associated with the selected RSFCs. The estimated and chronological MoCA scores were significantly correlated with *R* = 0.81 and the mean absolute error (MAE) = 1.20. Additionally, cognitive impairments of patients with T2DM can be identified using the RSFC pattern with classification accuracy of 90.54% and the area under the receiver operating characteristic (ROC) curve (AUC) of 0.9737. This connectivity pattern not only included the connections between regions within the default mode network (DMN), but also the functional connectivity between the task-positive networks and the DMN, as well as those within the task-positive networks. The results suggest that an RSFC pattern could be regarded as a potential biomarker to identify the cognitive status of patients with T2DM.

## Introduction

Type 2 diabetes mellitus (T2DM) is typically accompanied by cognitive impairments and is associated with a much higher risk of dementia [1], [2]. Patients with this disorder may experience a deterioration of memory, attention, information processing speed, and executive function [1], [3]. An in-depth understanding of the causative neuro-mechanism of cognitive impairment in the early stages of T2DM could help clinicians to identify patients with a high risk of dementia, and to consequently introduce effective interventions to retard and even arrest the progression of subtle cognitive decrements [4]. However, the mechanism behind the cognitive impairments in patients with T2DM remains unclear. Recently, an increasing number of neuroimaging studies on patients with T2DM revealed alterations in the gray matter volume and white matter integrity associated with cognitive impairment [5], [6]. This suggests that changes in the neural substrates of patients with T2DM are likely linked to cognitive dysfunction.

Resting state functional connections (RSFCs) are typically used in neuroimaging studies to measure the correlation between the fMRI time series of different brain regions without any external disturbance, they also can reflect on certain intrinsic mechanisms in the human brain [7]. The whole brain RSFCs are a cumulation of the connections of all paired regions of our brain and are relatively rich in information relevant to the intrinsic interactions among the regions of the brain that are induced by the spontaneous neural activities. Therefore, an analysis of the connectivity patterns based on the whole brain RSFCs, in comparison with the techniques based on individual connectivity, could provide us a much more comprehensive understanding on the neural mechanism of certain cognitive disorders. Whole brain RSFCs have previously been used in a number of studies addressing cognitive disorders. Previous studies have reported that patients with Alzheimer’s disease (AD) or mild cognitive impairment (MCI) had abnormal connectivity patterns [8], [9]. Whole brain RSFCs have also been used to successfully select biomarkers in recent fMRI studies, such as age estimation [10] and identification of psychiatric disorders [11]. Thus, it can be speculated that some of connectivity patterns could be regarded as potential biomarkers to either evaluate or identify cognitive impairment in patients with T2DM.

Recently, a number of RSFC-based studies investigated differences between patients with T2DM and normal controls using their neural substrate and some have reported abnormal RSFCs in their default mode network (DMN) [12], [13], [14]. Yang et al. had particularly [14] reported abnormalities in the connectivity within the DMN along with those among other RSFC networks in patients with T2DM related cognitive impairment. Another resting state study demonstrated a decrease in RSFCs associated with the attention network of patients with T2DM and cognitive impairment [15]. In addition to RSFC, Cui et al. [16] reported that the amplitude of low frequency fluctuation and regional homogeneity, which were both calculated from the resting state brain activity, changed in regions of DMN and other brain regions, such as the visual and auditory network of patients with T2DM and cognitive impairment. These findings suggested that the whole brain RSFCs provided more information about the mechanism of T2DM-related cognitive impairment. However, these studies focused on the difference in individual connectivity at a group level between normal controls and patients with T2DM, and did not directly examine the relationship between the whole brain RSFC patterns and cognitive status in patients with T2DM. Thus, these neural substrate findings cannot be used in clinical practice for quantitatively evaluate the progression of cognitive impairment in patients with T2DM.

To bridge this gap, the present study used a multi-variate pattern analysis (MVPA), which has been typically used in neuroimaging studies and demonstrated to be an effective method to analyze fMRI data [10], [11], [17] and examine the correlations between cognitive status of patients with T2DM and whole brain RSFCs. We particularly aimed to identify potential biomarkers that could be used to detect patients with T2DM, who have a high risk of presenting cognitive impairment.

We first constructed a predictive model with whole brain RSFCs serving as predictors and the Montreal Cognitive Assessment (MoCA), which can be used to measure the degree of general cognition of all participants (regardless of cognitive impairment) serving as the dependent variable. However, whole brain RSFCs include a large number of predictors, which is significantly larger than the number of participants (*i.e.*, the number of measurement). Furthermore, there are a number of correlations among features of whole brain RSFCs, making it difficult to estimate a model while utilizing an ordinary regressing strategy such as general linear modeling. To address these problems, we used an elastic net (E-Net) to construct a regression model to reduce the dimensions of the features. The E-Net can both successfully select an appropriate feature and estimate a model by balancing the goodness of fit and model complexity and compensating for the correlations among the whole brain RSFC features [18].

The surviving predictors after estimating the E-Net model, namely the selected features of whole brain RSFCs, are the potential biomarkers to evaluate the MoCA scores of participants. To further validate the roles of these selected features, a machine learning method was used to evaluate their discriminative ability. This step can not only evaluate the diagnostic valuation of these features, but also help in revealing the neural mechanism behind cognitive impairment in patients with T2DM.

## Results and discussion

### Characteristics of patients

The characteristics of the enrolled patients are shown in Table 1. The average age of all patients was 54.51 years and 68.4% patients were men (65 of 95). Moreover, the average MoCA score of the patients was 25.85, which was similar to normal cognition. Functional MRI data of the 95 patients enrolled, were preprocessed and we performed a functional connectivity analysis to detect whole brain RSFCs for all the patients. Subsequently, functional connectivity between two of the 90 brain regions were obtained for every patient. Thus, we identified 4005 features for each patient with T2DM for further analysis.

**Table 1:** **Characteristics of the T2DM patients examined in this study**

**Characteristics**	**T2DM patients (n = 95)**
Age (year)	54.51 ± 8.90
Sex (male/female)	65/30
Fasting glucose (mM)	8.97 ± 2.58
HbA_1c_ % (mmol/mol)	8.24 ± 1.71 (66.5 ± 18.7)
Total cholesterol (mM)	4.51 ± 1.08
BMI (kg/m^2^)	25.31 ± 3.15
Disease duration (year)	9.57 ± 6.33
MoCA	25.85 ± 1.97

*Note*: Data are presented as mean ± SD. HbA_1c_, glycated hemoglobin; BMI, body mass index; MoCA, Montreal Cognitive Assessment; SD, standard deviation.

### Cognition estimation and selection of key RSFCs

**Figure 1:**
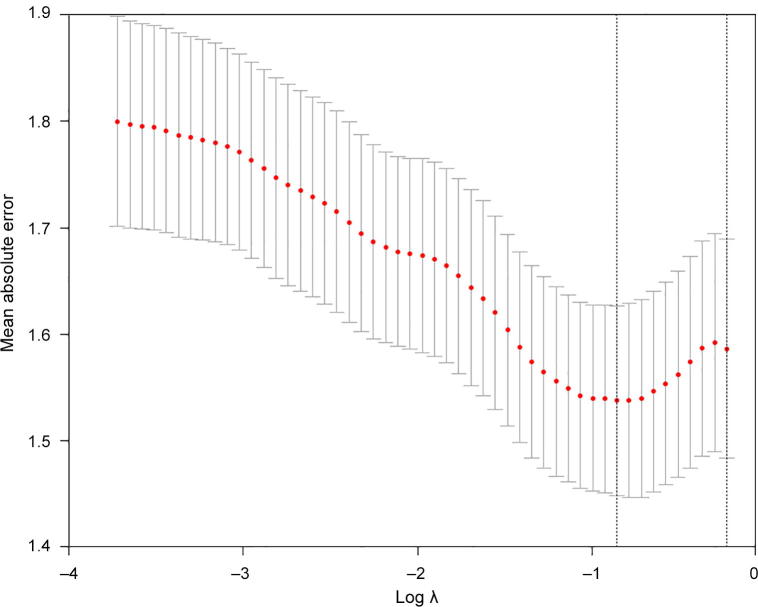
**Feature selection with E-net** Tuning parameter selection in the E-net model used 10-fold cross-validation via minimum criteria. The mean absolute error is plotted versus log (*λ*). The left vertical line represents the value of *λ* that gives minimum mean absolute error, and the right vertical line represents the largest value of *λ* that error was within 1× standard error of the minimum. The left vertical line was used as the optimal value in this study.

**Figure 2:**
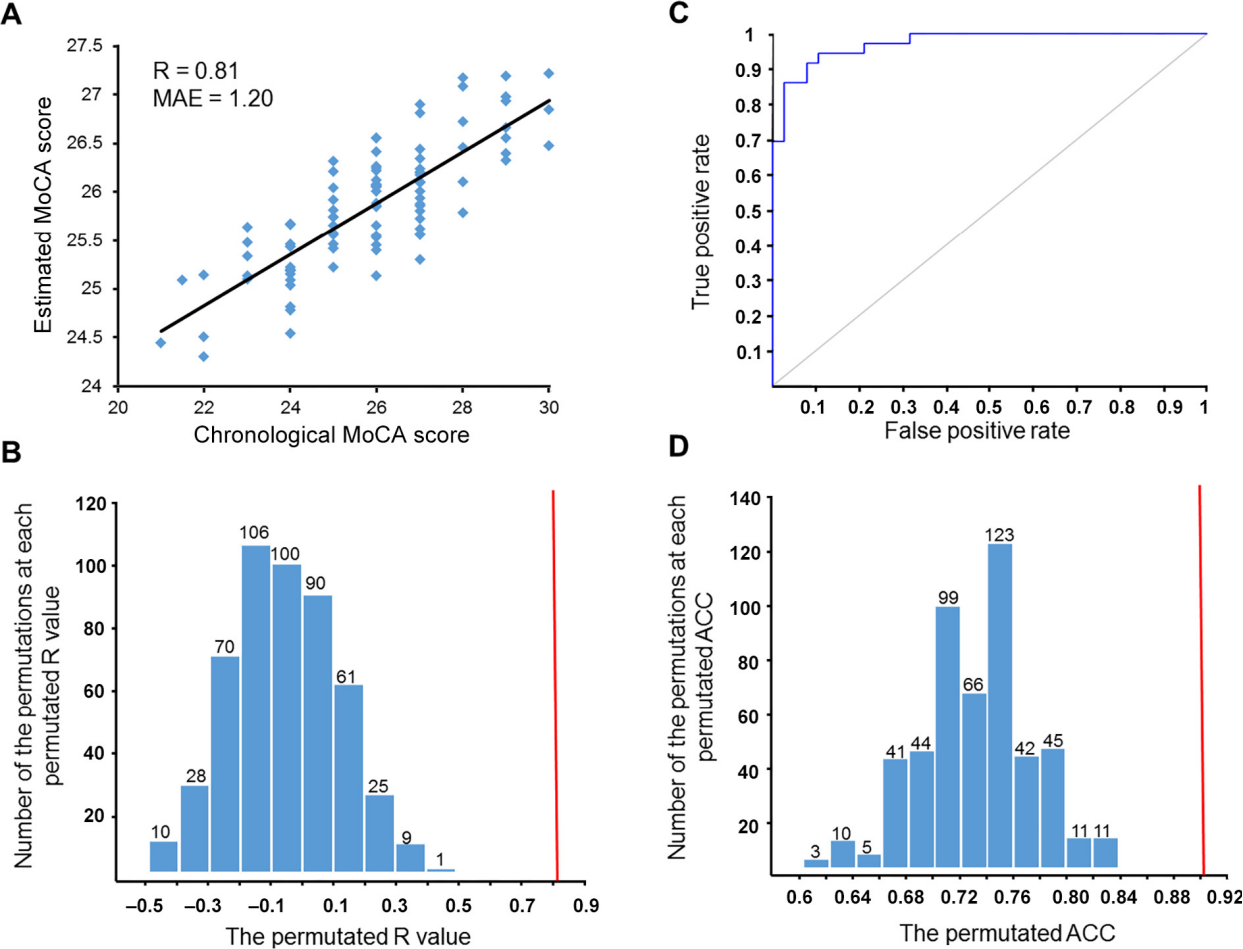
**Results of MoCA estimation and cognition classification** **A.** The plot of real versus estimated MoCA scores. **B.** The result of the permutation test to estimate MoCA. The predictions based on 500 permutations were evaluated by the Pearson's correlation coefficients between the estimated and permuted MoCA. The red line indicates the estimation based on non-permuted MoCA. **C.** The ROC curve to classify cognition (AUC = 0.9737). The estimated MoCA was calculated by the E-net model, and the real MoCA was obtained by a MoCA test. **D.** The result of the permutation test to classify cognition. Predictions were based on 500 permutations, and the classifications were evaluated by the classification rates. The red line indicates the classification rate based on non-permuted labels. MoCA, Montreal Cognitive Assessment; ACC, accuracy; MAE, mean absolute error; ROC, receiver operating characteristic; AUC, the area under the ROC curve.

To explore the relationship between cognition of patients with T2DM and whole brain RSFCs, we first constructed a cognition estimation model to reveal the association between the MoCA score and whole brain RSFCs. Considering the limited sample size in comparison with the number of RSFCs, we aimed to reduce the latter used in the cognition prediction model to avoid a potential overfitting. Since each RSFC was not directly correlated with a MoCA score, we utilized an E-net to obtain the most valuable RSFCs to estimate the MoCA. E-net was a very useful tool as it could reduce the dimensionality of features, and concurrently isolate key features. We used a 10-fold cross-validation via minimum criteria to select the tuning parameter in the E-net model (Figure 1). The best MoCA estimated model was detected as *α* = 0.9 and *λ* = 0.424. The results of the MoCA estimation revealed an observable association between the MoCA score and whole brain RSFCs. It showed that, the estimated MoCA scores and real MoCA scores was significantly correlated with *R* = 0.81 (Figure 2) and *P* = 0.002. The results indicated that there was no better prediction in the 500 permutations (Figure 2). Additionally, the MAE between the estimated and real MoCA score was 1.20 (Figure 2), suggesting that the cognitive decline of patients with T2DM that was indexed by MoCA scores could be predicted by a combination of some RSFCs (*i.e.*, connectivity pattern) with a relatively good performance.

**Figure 3:**
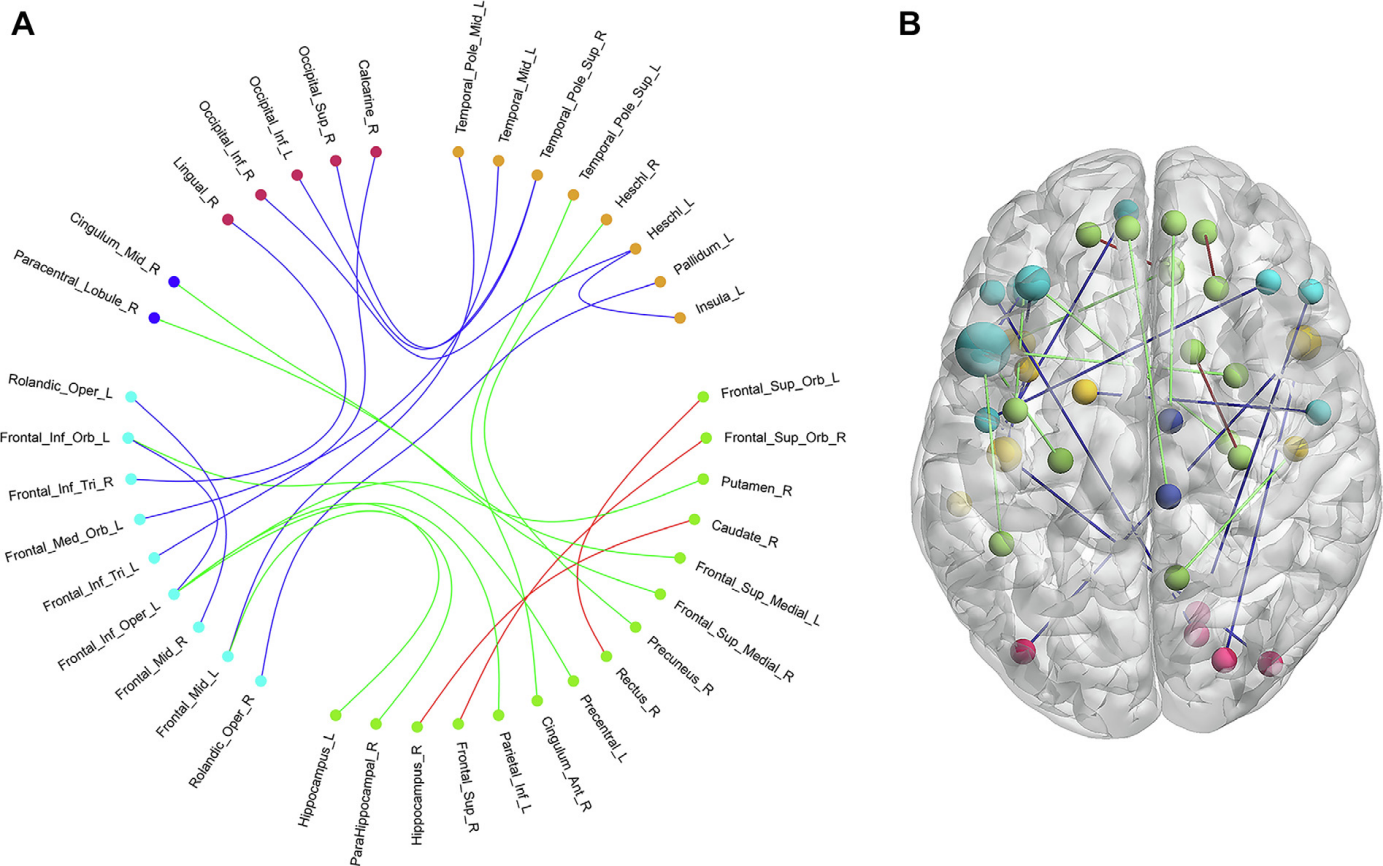
**The RSFCs selected from the MoCA estimation model** **A.** The network connectivity diagram of the RSFCs selected from the MoCA estimation model. **B.** Brain surface rendering of the RSFCs selected from the MoCA estimation model. The orange dots indicate the brain regions of auditory network and the green dots indicate brain regions of DMN, the indigo dots indicate the brain regions of effective control network, the blue dots indicate the brain regions of motor network, and the red dots indicate the brain regions of visual network. The red lines indicate the RSFCs between regions of the DMN, the green lines indicate the RSFCs between regions of the DMN and regions of task-positive networks, and the blue lines indicate the RSFCs between regions of task-positive networks. In panel B, larger dots indicate more connections of the brain regions. DMN, default mode network; RSFC, resting state functional connection.

We selected a connectivity pattern consisting of 23 RSFCs using a non-zero coefficient in the best MoCA scores estimation model corresponding to greater contributions to the estimation of the MoCA scores. We did not include any clinical characteristics in the best model. The selected 23 RSFCs, as key features of the MoCA estimation, are illustrated in Figure 3, and detailed information about the same, such as their correlations with MoCA as well as their contributions to the classification of cognition is listed in Table 2. As indicated in Table 2, the selected connectivity pattern mainly consisted of RSFCs within the DMN, followed by between the DMN and other resting state brain networks such as the audio network (AN), the visual network (VN), effective control network (ECN), and the motion network (MN). These findings suggest that the cognitive impairment of patients with T2DM may be associated with the abnormality of connectivity with and between these different resting state networks.

**Table 2:** **RSFCs selected as the key features from the MoCA estimation model for cognition classification**

**ROI1**			**ROI2**			**Contribution**	**Correlation with MoCA**	***P* value**
**Brain region**	**Coordinate**	**RSN**	**Brain region**	**Coordinate**	**RSN**			
CAU_R (25)	11, 18, 5	DMN	HIP_R (20)	25, −10, −15	DMN	−0.20651	−0.297	0.069
SFG_orb_R (11)	22, 63, −6	DMN	SFG_R (6)	22, 0, 64	DMN	0.00917	0.349	0.023*
REC_R (11)	8, 35, 18	DMN	SFG_orb_L (11)	−16, 47, −13	DMN	−0.07694	−0.262	0.23
IFG_orb_L (47)	−40, 36, −12	ECN	IFG_oper_L (48)	−53, 14, 9	ECN	0.158143	0.338	0.023*
IPG_L (40)	−45, −43, 53	DMN	IFG_oper_L (48)	−53, 14, 9	ECN	0.157015	0.327	0.023*
HIP_L (20)	−25, −21, −10	DMN	IFG_oper_L (48)	−53, 14, 9	ECN	0.079794	−0.261	0.253
HES_L (48)	−46, −15, 12	AN	INS_L (48)	−40, 17, −2	AN	0.036246	−0.305	0.069
STGp_L (38)	−40, 15, −20	AN	ACC_R (24)	8, 37, 16	DMN	−0.0138	0.263	0.23
IFG_orb_L (47)	−40, 36, −12	ECN	PreCG_L (6)	−42, 0, 32	DMN	−0.03047	0.347	0.023*
HES_L (48)	−46, −15, 12	AN	IOG_R (19)	38, −81, −7	VN	−0.06352	0.225	0.667
LING_R (18)	16, −67, −3.87	VN	IFG_tri_R (45)	50, 30, 14	ECN	−0.06527	−0.297	0.069
MCC_R (24)	8, −9, 40	MoN	SFG_med_R (10)	9, 51, 30	DMN	−0.06985	−0.279	0.138
CAL_R (17)	17, −68, 10	VN	IFG_tri_L (45)	−48, 35, 12	ECN	−0.07421	−0.365	0.023*
HES_R (48)	46, −15, 12	AN	PCNU_R (5/23)	6, −57, 59	DMN	−0.07937	0.321	0.046*
STGp_R (38)	54, 9, −2	AN	IOG_L (19)	−36, −82, −8	VN	−0.11025	−0.349	0.023*
ROL_oper_L (48)	−47, −8, 14	ECN	MFG_R (8)	37, 33, 34	ECN	−0.13138	−0.262	0.23
PUT_R (48)	29, 6, 8	DMN	IFG_oper_L (48)	−53, 14, 9	ECN	−0.13228	−0.292	0.092
MTG_L (21)	−54, −54, 8	AN	MFG_orb_L (10)	−5, 51, −6	ECN	−0.17921	−0.307	0.046*
PAL_L (48)	−18, 0, 0.21	AN	ROL_oper _R (48)	53, −6, 15	ECN	−0.18165	−0.239	0.46
MTGp_L (38)	−43, 16, −32	AN	MFG_L (8)	−33, 10, 54	ECN	−0.21036	−0.348	0.023*
STGp_R (38)	54, 9, −2	AN	SOG_R (19)	23, −76, 34	VN	−0.22058	−0.296	0.092
PCL_R (4)	7, −32, 68	MoN	SFG_med_L (10)	−5, 49, 31	DMN	−0.23836	−0.262	0.23
PHG_R (28)	25, −15, −20	DMN	MFG_L (8)	−33, 10, 54	ECN	−0.28274	−0.251	0.322

*Note*: RSFC, resting state functional connection; ROI; region of interest; RSN, resting state network; DMN, default mode network; ECN, executive control network, AN, auditory network; MoN, motor network; VN, visual network. CAL, calcarine; CAU, caudate; ACC, anterior cingulum cortex; MCC, middle cingulum cortex; HES, heschl; HIP, hippocampus; IFG_oper, inferior frontal gyrus opercular; IFG_orb, IFG orbital; IFG_tri, IFG triangular; INS, insula; IOG, inferior occipital gyrus; IPG, inferior parietal gyrus; LING, lingual; MFG, middle frontal gyrus; MFG_orb, MFG orbital; MTG, middle temporal gyrus; MTGp, MTG pole; PAL, pallidum; PCL, paracentral lobule; PHG, parahippocampal gyrus; PreCG, precentral gyrus; PCNU, precuneus; PUT, putamen; REC, rectus; ROL_oper, rolandic opercular; SFG, superior frontal gyrus; SFG_med, SFG medial; SFG_orb, SFG orbital; SOG, superior occipital gyrus; STGp, superior temporal gyrus pole; R, right; L, left. *, *P <* 0.05 (after Bonferroni correction for the number of features).

Among the selected 23 RSFCs, there were 3 between the brain regions within DMN (Figure 3). The first was the functional connection between the right caudate and the right hippocampus. The second was the connectivity between the right superior frontal gyrus (orbital) and the right superior frontal gyrus. The third was the connectivity between the right rectus and the left superior frontal gyrus (orbital). DMN is considered to be a major contributor to cognitive function [19], [20], and the abnormal activity of the DMN has been demonstrated to be related to some mental disorder such as MCI [21], AD [22] or other mental disorders [23]. Particularly, Yang et al. [14] found that cognitive impairment in patients with T2DM presented a significantly decreased RSFC strength within the DMN than the normal controls; however, such an abnormality was not observed when comparing patients with T2DM who have normal cognition and normal controls. This evidence along with our findings suggested that the cognitive impairment of patient with T2DM might be related to the normality of connections within the DMN.

Among the 23 selected key RSFCs, there were 9 between regions of DMN and regions of task-positive networks (Figure 3, Table 2). First, there were 5 connections between the DMN and the ECN, namely the ones between the left hippocampus and left inferior frontal gyrus (opercular), the left precentral gyrus and left inferior frontal gyrus (orbital), the right putamen and left inferior frontal gyrus (opercular), the left inferior parietal gyrus and left inferior frontal gyrus (opercular), and between the right parahippocampal gyrus and left middle frontal gyrus. Second, there were two connections between the DMN and the AN, namely the ones between the right anterior cingulum cortex and left superior temporal gyrus (pole), and between the right precuneus and right heschl. Lastly, there were two connections between the DMN and the MoN, namely the ones between the right superior frontal gyrus (medial) and right middle cingulum cortex, and between the left superior frontal gyrus (medial) and right paracentral lobule.

The present study also revealed the connections among task-positive networks (Figure 3, Table 2). There were 3 connections between the AN and the ECN, 3 connections between the AN and the VN, and 2 connections between the VN and ECN. Additionally, there were 2 connections within the ECN and 1 connection within the AN. The results suggested that hippocampus could be a crucial central hub in the selected RSFCs, which was consistent with findings of previous studies focusing on the insulin resistance in youth [24], [25], [26]. Although the dysconnectivity patterns in hippocampal and striatal reward regions in youth susceptible to diabetes relates directly to the degree of insulin resistance, our finding further suggested that RSFCs related with hippocampus were significantly correlated with the cognitive impairments in patients with T2DM.

### Cognition classification and performance evaluation

**Figure 4:**
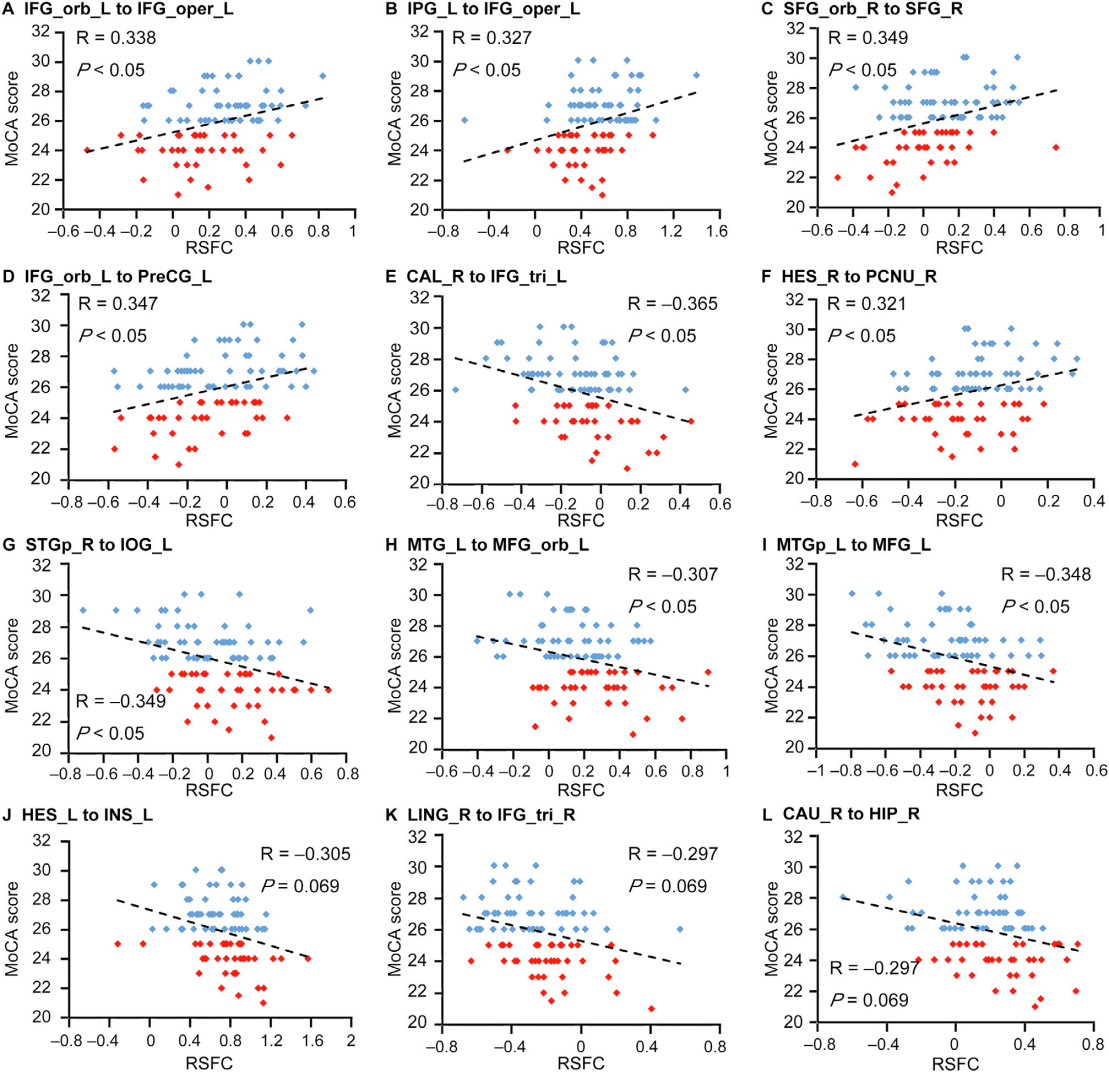
**Correlations between the selected RSFCs and MoCA scores** Pearson’s correlations between the selected RSFCs and MoCA scores were calculated for different region pairs. The red dots indicate the cognition impairment group with MoCA scores <26. The blue dots indicated the normal cognition group with MoCA scores >26. The dashed line indicates the correlation trend line. IFG_orb_L, left inferior frontal gyrus orbital; IFG_oper_L, left inferior frontal gyrus opercular; IFG_tri_L, left inferior frontal gyrus triangular; IPG_L, left inferior parietal gyrus; SFG_orb_R, right superior frontal gyrus orbital; SFG_R, right superior frontal gyrus; PreCG_L, left precentral gyrus; CAL_R, right calcarine; HES_R, right heschl; PCNU_R, right precuneus; STGp_R, right superior temporal gyrus pole; IOG_L, left inferior occipital gyrus; MTG_L, left middle temporal gyrus; MFG_orb_L, left middle frontal gyrus orbital; MTGp_L, left middle temporal gyrus pole; MFG_L, left middle frontal gyrus; CAU_R, right caudate; HIP_R, right hippocampus; LING_R, right lingual; INS_L, left insula.

**Figure 5:**
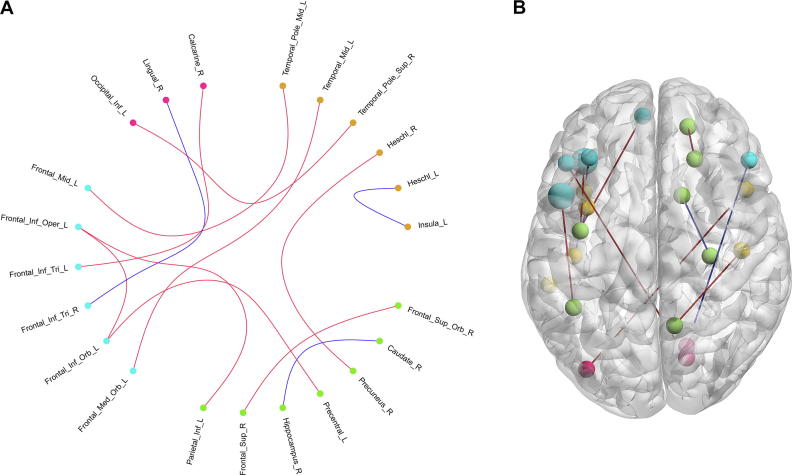
**RSFCs demonstrate significant or marginally significant correlations with MoCA scores** **A.** The network connectivity diagram of the RSFCs which were significantly or marginally significantly associated with MoCA scores. **B.** Brain surface rendering of the RSFCs demonstrated significantly or marginally significantly associated with MoCA scores. The red lines indicate the RSFCs significantly correlated with MoCA scores, the blue lines indicate the RSFCs showed marginally significant correlations with MoCA scores. The orange dots indicate the brain regions of auditory network, the green dots indicate the brain regions of DMN, the indigo dots indicate the brain regions of effective control network, and the red dots indicate the brain regions of visual network. In panel B, larger dots indicate more connections of the brain regions. Associations between RSFCs and MoCA scores are considered significant with *P* < 0.05 after Bonferroni correction for the number of features, while associations are considered marginally significant with *P* ≈ 0.05 after Bonferroni correction for the number of features,.

**Figure 6:**
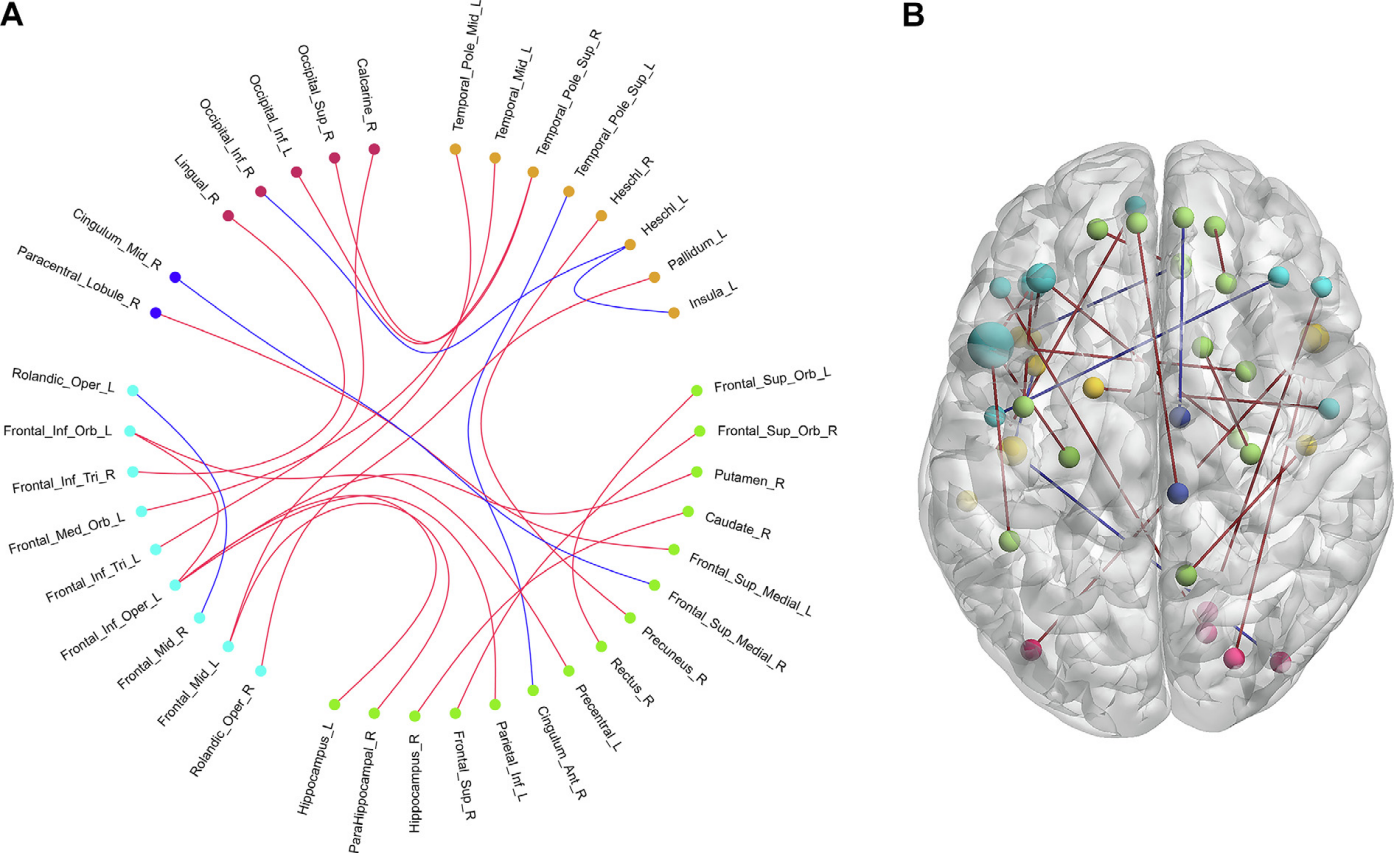
**RSFCs comparison between normal cognition group and cognition impairment group** **A.** The network connectivity diagram of the RSFC comparison between normal cognition group and cognition impairment group. **B.** Brain surface rendering of the RSFCs comparison between normal cognition group and cognition impairment group. The orange dots indicate the brain regions of auditory network, the green dots indicate the brain regions of DMN, the indigo dots indicate the brain regions of effective control network, the blue dots indicate the brain regions of motor network, and the red dots indicate the brain regions of visual network. The red lines indicate significant difference in RSFCs between the two groups, whereas the blue lines indicate that there is no significant difference in RSFCs between the two groups. In panel B, larger dots indicate more connections of the brain regions. Differences in RSFCs between normal cognition group and cognition impairment group are considered significant with *P* < 0.05 (two sample *t* test).

We conducted a correlation analysis between the selected features and MoCA scores to investigate the relationship of these selected RSFCs to the cognitive performance. Seven of the selected 23 RSFCs demonstrated significant positive correlation with MoCA scores (*r* > 0, and *P < *0.05 after Bonferroni correction for the number of features), and two of the selected 23 RSFCs showed a significant negative correlation with MoCA scores (*r* > 0, and *P < *0.05 after Bonferroni correction for the number of features). Additionally, there were three RSFCs that showed marginally significant negative correlations with MoCA scores. Detailed information regarding the correlation analysis is shown in Figure 4. Moreover, these RSFCs were presented in Figure 5. We can see that five of the nine RSFCs with significant or marginally significant correlations with MoCA scores involved in brain regions of DMN, suggesting the importance of DMN. To investigate if there were differences between the patients with normal and impaired cognition, a two-sampled t-test of the selected 23 RSFCs were performed between the two groups (Figure 6). Most of these RSFCs (18/23) showed significant differences between these two groups (*P <* 0.05), while all of the 5 RSFCs without significant differences were either between regions of DMN and regions of task-positive networks or among the regions of task-positive networks.

Classification of the results of cognition indicated that the SVM classifier achieved a performance with an AUC of 0.9737 and the accuracy of classification was 90.54%, based on the selected 23 RSFCs while using a 10-fold cross validation (Figure 2), and there was an absence of better classification results that were detected using the non-parametric permutations with a *P* value of *P* = 0.002 (Figure 2). Additionally, the sensitivity and specificity of the classification model were 92.11% and 88.89%, respectively. The contribution of each selected RSFC to the classification of cognition was analyzed with its weight in the SVM model. Detailed information about the contributions of the 23 RSFCs is provided in Table 2.

The relatively high sensitivity and specificity of the classification model ensured that the 23 RSFCs selected via E-net analysis could be used as the potential biomarkers to either evaluate or identify the cognitive impairment of T2DM patients. It was noted that, as revealed via correlation analysis, more than half of the 23 RSFCs (14 out of 23) were not significantly correlated with the MoCA score. Therefore, the single-variable method (*e.g.*, Pearson correlation) “underestimates” the role of these RSFCs in evaluating the cognitive decline of patients with T2DM. Furthermore, due to the limited number of brain regions and connectivity between these regions, the connectivity patterns do not individually match with a large number of cognitive processes. Therefore, according to a network theory, an interaction among different regions or networks may be more relevant to a certain cognitive process than any particular individual region or network [22], [27], [28]. If this idea is correct, the RSFC pattern that we identified using a MVPA may provide a relatively complete profile of the neural mechanism of cognitive impairment in patients with T2DM, and reveal potential biomarkers to evaluate or identify the associated cognitive impairment.

### Limitations

The present study has a number of limitations. First, it was a cross-sectional investigation that only focused on the cognitive impairment in patients with T2DM. However, patients with T2DM and cognitive impairment are at a significant risk of developing AD and MCI; therefore, further longitudinal studies are required to elucidate the changes in the whole brain RSFCs with regard to this cognitive impairment, particularly as it progresses into either AD or MCI. Additionally, although the sample size in the present study was large for an fMRI study, it was relatively small for a clinical study, and this is also a reason to explain the absence of independent validation. Future studies with a larger population sample or multicenter imaging data may further confirm the potentiality of the RSFCs in predicting the cognitive impairment in patients with T2DM, and develop an outperformed prediction model with independent validation. Lastly, possible effects of different hypoglycemic agents may impact the results. Future studies should take this into consideration and explore its potential effects.

## Conclusion

Here we used an MVPA method to investigate the correlations between the RSFCs and cognitive impairments in patients with T2DM. We observed that the T2DM associated cognitive decline could be predicted via an RSFC pattern. This connectivity pattern included the connections between regions within the DMN, along with those between the task-positive networks and the DMN, as well as those within the task-positive networks. Although majority of these connections do not individually demonstrate significant correlation with the cognition of patients with T2DM, all of them together are significantly correlated with cognitive impairment. These results suggest that this RSFC pattern may play important roles in the T2DM related cognitive decline; therefore, can be regarded as potential biomarkers to evaluate or identify this cognitive impairment.

## Materials and methods

### Patients

We recruited 95 patients with T2DM from the Henan Provincial People’s Hospital. We used the newest criteria of the American Diabetes Association [29] to define the disease. All these patients were between 43 and 75 years of age, had been suffering from the disease for longer than a year, were routinely treated with hypoglycemic agents, and were closely self-monitored. Our exclusion criteria were as follows: (1) a history of brain lesions such as stroke or brain tumors; (2) a history of alcohol or substance abuse; (3) a psychiatric or neurological disease, including major depression or any other psychopathology; (4) a history of hypertension; (5) a history of hypoglycemic episodes; and (6) contraindications to MRI. All patients underwent a MoCA test to examine their general cognition [30]; an education-adjusted MoCA score was detected for every patient [31]. The Ethics Committee of Henan Provincial People’s Hospital approved the present study in accordance with the Helsinki declaration, and written informed consent was obtained from all patients prior to their participation in our study. The characteristics of the patients are summarized in Table 1.

### Data acquisition and imaging processing

All MRI images were captured using a Magnetom Trio 3.0 T scanner (Siemens, Erlangen, Germany) at the Radiology Department of Henan Provincial People’s Hospital utilizing a 12-channel receive-only head coil. The patients were instructed simply to rest with their eyes closed, to relax, but not fall asleep, and to keep still. Functional resting state images were acquired using a gradient echo T2-weighted pulse sequence with TR = 2000 ms, TE = 30 ms, matrix = 64 × 64, field of view (FOV) = 240 mm × 240 mm, thickness = 4 mm, and flip angle = 90°. The functional resting state scan lasted for 7 min, and 210 volumes were collected.

The data was preprocessed via the Data Processing Assistant for Resting-State toolbox (DPARSF; http://www.restfmri.net/forum/DPARSF). We discarded the first ten volumes to equilibrate the magnetic field. All remaining volumes were then realigned to adjust for head motions using a least-squares minimization technique. Any patient with head motion >2.0 mm of translation or >2.0° of rotation in any direction were excluded. We then further processed the image data with the spatial normalization based on Montreal Neurological Institute (MNI) space [32], and resampled to 3 × 3 × 3 mm^3^. Smoothing (full width at half maximum [FWHM] = 4 mm), detrending, and filtering (0.01–0.08 Hz) were performed in order. Regression analysis including six head motion parameters and mean time series of global, white matter, and cerebrospinal fluid signals were conducted to remove the possible effects on the results.

We performed functional connectivity analysis using Resting-State fMRI Data Analysis Toolkit (REST). The registered images were divided into 90 regions according to the automated anatomical labeling atlas [33]. The atlas divided the cerebrum into 90 regions (45 in each hemisphere). The representative time series of each region used to analyze functional connectivity was estimated by averaging the fMRI time series over all voxels in the region. We evaluated the functional connectivity between each pair of regions with a Pearson correlation coefficient. A Fisher’s transform was applied to improve the normality of the correlations. Therefore, a resting-state functional network captured via a 90 × 90 symmetric matrix was detected for each patient. We removed 90 diagonal elements and the upper triangle elements of the matrix were used as features for further analysis, *i.e.*, the feature space was spanned by the (90 × 89)/2 = 4005-dimensional feature vectors.

### MoCA estimation and feature selection

The MoCA scores were estimated with a linear model based on the 4005 RSFCs and the clinical characteristics of patients including their age, sex, fasting glucose, glycated hemoglobin (HbA_1c_), total cholesterol, body mass index (BMI), and disease duration. The linear model is defined as follows:

$$y=\sum_{i=1}^{n}\beta_{i}x_{i}+\beta_{0}+\varepsilon$$

Where, *n* was the number of features used in the model, here *n* = 4012; *y* was MoCA score of the patients; $x_{i}$ (*i* = 1, 2, …, *n*) was the predicting parameter in the model, such as the RSFC or its clinical characteristics; $\beta_{i}$ (*i* = 0, 1, 2, …, *n*) was coefficient of each parameter, and ε was the error term. E-Net was used to estimate the coefficients of the model [34]. Here, we simultaneously estimated the coefficients and selected the feature, minimizing the following cost function:

$$\sum_{i=1}^{N}{\left(y_{i}-\sum_{j=1}^{n}\beta_{j}x_{\mathrm{ij}}-\beta_{0}\right)}^{2}+\lambda \sum_{j=1}^{n}\left(\alpha \left|\beta_{j}\right|+0.5\left(1-\alpha \right){\left(\beta_{j}\right)}^{2}\right)$$

Where, *N* was the number of patients; *x_ij_* was the *j*th feature of the *i*th patients; *y_i_* was the MoCA score of the *i*th patients; and *λ* and *α* were regularization parameters. Subsequently, features with a greater contribution to the MoCA estimation could be selected.

We used glmnet [35] to estimate MoCA. We selected *λ* and *α* and estimated MoCA using a 10-fold cross validation. The candidate values for the *λ* and *α* parameters were both restricted to a range between 0 and 1 in steps of 0.1, in which the optimal *λ* and *α* were selected via minimum criteria. The model goodness criteria that we employed were the MAE and the correlation between the estimated and real MoCA scores. The features of the best estimation model were further used to classify cognition.

### Cognition classification and performance evaluation

To classify cognition, 36 patients with a MoCA score >26 were taken as the normal cognition group, while 38 patients with a MoCA score <26 were taken as the cognition impairment group. We classified cognition for these two groups with the features selected from the MoCA estimation model. When we obtained the data for features that highly correlated with MoCA, we used linear support vector machines (SVM) to perform cognition classification. The toolbox LibSVM [36] was used to construct the classification model based on the selected features. The results were based on the best parameter setting. We then investigated the discrimination performance of the SVM model with a 10-fold cross validation. The classification results were further interpreted using the classification accuracy, specificity, and sensitivity based on the cross-validation. Moreover, the ROC curve and AUC were also calculated.

### Evaluation of the selected RSFCs

We performed a correlation analysis between the selected RSFCs and MoCA scores to investigate the relationship of these selected RSFCs to the cognitive performance. A linear regression was identified between the RSFC and MoCA score of each included patient, so we can detect the correlation between them.

We also analyzed the contribution of each RSFC to the cognitive classification. The weight value of the RSFC in the SVM model was considered as contribution to classify cognition. The weight value of each feature was calculated through summing the coefficients allocated to the RSFC across the 10-fold cross validation, and then normalized by dividing the maximal coefficients across all the selected features.

### Permutation tests

The framework of permutation tests to assess predictive performance has been used in several previous studies [10], [11]. We performed 500 permutations to estimate the probability of detecting identical MoCA estimation/classification performance. Specifically, the MoCA (for MoCA estimation)/cognition labels (for the classification of cognition) of the patients were permuted 500 times randomly. Furthermore, the *P* value of the probability for detecting the estimation/classification accuracy was defined as follows:

$$P=\frac{1+N_{\mathrm{Better}\;predictions}}{1+N}$$

Where, *N* = 500 here represents the number of permutations; to estimate MoCA, *N_Better predictions_* was the number of permutations with larger correlations (compared to the result based on non-permuted MoCA scores) between the permuted and estimated MoCA scores for the estimation; and to classify cognition, *N_Better predictions_* was the number of permutations with higher classification accuracy (compared to the result based on non-permuted labels).

## Data availability

Please contact with the corresponding authors for data access. The MRI image data will be available upon request after approval by the Ethics Committee of Henan Provincial People’s Hospital, China.

## Authors’ contributions

HY, TL, and MW collected the data, MW, YL, and JT contributed to the study design and revised the manuscript for intellectual content, XC, ZT, and YD performed the analysis, ZL and JL wrote the manuscript. JT is the guarantor of this manuscript, has full access to all the data in the study, and takes responsibility for the integrity of the data and the accuracy of the data analysis. All authors have read and approved the final manuscript.

## Competing interests

The authors have declared no competing interests.

## References

[1] Kodl C.T., Seaquist E.R. (2008). Cognitive dysfunction and diabetes mellitus. Endocr Rev.

[2] McCrimmon R.J., Ryan C.M., Frier B.M. (2012). Diabetes and cognitive dysfunction. Lancet.

[3] Biessels G.J., Strachan M.W.J., Visseren F.L.J., Kappelle L.J., Whitmer R.A. (2014). Dementia and cognitive decline in type 2 diabetes and prediabetic stages: towards targeted interventions. Lancet Diabetes Endocrinol.

[4] Biessels G.J., Reijmer Y.D. (2014). Brain changes underlying cognitive dysfunction in diabetes: what can we learn from MRI?. Diabetes.

[5] Zhang J.Y., Wang Y.X., Wang J., Zhou X.Q., Shu N., Wang Y.Y. (2014). White matter integrity disruptions associated with cognitive impairments in type 2 diabetic patients. Diabetes.

[6] Zhang Y.W., Zhang X., Zhang J.Q., Liu C., Yuan Q.Y., Yin X.T. (2014). Gray matter volume abnormalities in type 2 diabetes mellitus with and without mild cognitive impairment. Neurosci Lett.

[7] Fox M.D., Snyder A.Z., Vincent J.L., Corbetta M., Van Essen D.C., Raichle M.E. (2005). The human brain is intrinsically organized into dynamic, anticorrelated functional networks. Proc Natl Acad Sci U S A.

[8] Liu Z.Y., Zhang Y.M., Bai L.J., Yan H., Dai R.W., Zhong C.G. (2012). Investigation of the effective connectivity of resting state networks in Alzheimer's disease: a functional MRI study combining independent components analysis and multivariate granger causality analysis. NMR Biomed.

[9] Liu Z.Y., Zhang Y.M., Yan H., Bai L.J., Dai R.W., Wei W.J. (2012). Altered topological patterns of brain networks in mild cognitive impairment and Alzheimer's disease: a resting-state fMRI study. Psychiatry Res Neuroimaging.

[10] Tian L.X., Ma L., Wang L.L. (2016). Alterations of functional connectivities from early to middle adulthood: clues from multivariate pattern analysis of resting-state fMRI data. Neuroimage.

[11] Zeng L.L., Shen H., Liu L., Wang L.B., Li B.J., Fang P. (2012). Identifying major depression using whole-brain functional connectivity: a multivariate pattern analysis. Brain.

[12] Chen Y.C., Jiao Y., Cui Y., Shang S.A., Ding J., Feng Y. (2014). Aberrant brain functional connectivity related to insulin resistance in type 2 diabetes: a resting-state fMRI study. Diabetes Care.

[13] Cui Y., Jiao Y., Chen H.J., Ding J., Luo B., Peng C.Y. (2015). Aberrant functional connectivity of default-mode network in type 2 diabetes patients. Eur Radiol.

[14] Yang S.Q., Xu Z.P., Xiong Y., Zhan Y.F., Guo L.Y., Zhang S. (2016). Altered intranetwork and internetwork functional connectivity in type 2 diabetes mellitus with and without cognitive impairment. Sci Rep.

[15] Xia W.Q., Wang S.H., Rao H.Y., Spaeth A.M., Wang P., Yang Y. (2015). Disrupted resting-state attentional networks in T2DM patients. Sci Rep.

[16] Cui Y., Jiao Y., Chen Y.C., Wang K., Gao B., Wen S. (2014). Altered spontaneous brain activity in type 2 diabetes: a resting-state functional MRI study. Diabetes.

[17] Shen H., Wang L., Liu Y., Hu D. (2010). Discriminative analysis of resting-state functional connectivity patterns of schizophrenia using low dimensional embedding of fMRI. Neuroimage.

[18] Bunea F., She Y.Y., Ombao H., Gongvatana A., Devlin K., Cohen R. (2011). Penalized least squares regression methods and applications to neuroimaging. Neuroimage.

[19] Greicius M.D., Krasnow B., Reiss A.L., Menon V. (2003). Functional connectivity in the resting brain: a network analysis of the default mode hypothesis. Proc Natl Acad Sci U S A.

[20] Buckner R.L., Andrews-Hanna J.R., Schacter D.L. (2008). The brain's default network: anatomy, function, and relevance to disease. Ann NY Acad Sci.

[21] Zhan Y.F., Ma J.H., Alexander-Bloch A.F., Xu K.B., Cui Y., Feng Q.J. (2016). Liu Y, for the Alzheimer’s Disease Neuroimaging Initiative. Longitudinal study of impaired intra- and inter-network brain connectivity in subjects at high risk for Alzheimer's disease. J Alzheimers Dis.

[22] Uddin L.Q., Kelly A.M., Biswal B.B., Castellanos F.X., Milham M.P. (2009). Functional connectivity of default mode network components: correlation, anticorrelation, and causality. Hum Brain Mapp.

[23] Broyd S.J., Demanuele C., Debener S., Helps S.K., James C.J., Sonuga-Barke E.J. (2009). Default-mode brain dysfunction in mental disorders: a systematic review. Neurosci Biobehav Rev.

[24] Singh M.K., Leslie S.M., Packer M.M., Zaiko Y.V., Phillips O.R., Weisman E.F. (2019). Brain and behavioral correlates of insulin resistance in youth with depression and obesity. Horm Behav.

[25] Wroolie T.E., Kenna H.A., Singh M.K., Rasgon N.L. (2015). Association between insulin resistance and cognition in patients with depressive disorders: Exploratory analyses into age-specific effects. J Psychiatr Res.

[26] Watson K.T., Wroolie T.E., Tong G., Foland-Ross L.C., Frangou S., Singh M. (2019). Neural correlates of liraglutide effects in persons at risk for Alzheimer's disease. Behav Brain Res.

[27] Liao W., Mantini D., Zhang Z.Q., Pan Z.Y., Ding J.R., Gong Q.Y. (2010). Evaluating the effective connectivity of resting state networks using conditional Granger causality. Biol Cybern.

[28] McIntosh A.R. (2000). Towards a network theory of cognition. Neural Netw.

[29] American Diabetes A (2013). Diagnosis and classification of diabetes mellitus. Diabetes Care.

[30] Nasreddine Z.S., Phillips N.A., Bedirian V., Charbonneau S., Whitehead V., Collin I. (2005). The Montreal Cognitive Assessment, MoCA: a brief screening tool for mild cognitive impairment. J Am Geriatr Soc.

[31] Lu J., Li D., Li F., Zhou A.H., Wang F., Zuo X.M. (2011). Montreal Cognitive Assessment in detecting cognitive impairment in Chinese elderly individuals: a population-based study. J Geriatr Psychiatry Neurol.

[32] Ashburner J., Friston K.J. (1999). Nonlinear spatial normalization using basis functions. Hum Brain Mapp.

[33] Tzourio-Mazoyer N., Landeau B., Papathanassiou D., Crivello F., Etard O., Delcroix N. (2002). Automated anatomical labeling of activations in SPM using a macroscopic anatomical parcellation of the MNI MRI single-subject brain. Neuroimage.

[34] Zou H., Hastie T. (2005). Regularization and variable selection via the elastic net. J R Stat Soc Ser B-Stat Methodol.

[35] Friedman J., Hastie T., Tibshirani R. (2010). Regularization paths for generalized linear models via coordinate descent. J Stat Softw.

[36] Chang C.C., Lin C.J. (2011). LIBSVM: a library for support vector machines. ACM Trans Intell Syst Technol.

